# Supplementary material for: Investigating the upper bound of high-frequency electromagnetic waves on unshielded twisted copper pairs
Source: Nat Commun. 2022 Apr 26;13:2164. doi: 10.1038/s41467-022-29631-8 (PMC9043189; doi:10.1038/s41467-022-29631-8)
Supplement: Supplementary file 1 — Supplementary Information [file 41467_2022_29631_MOESM1_ESM.pdf]

Supplementary Information for “Investigating the Upper Bound of High-frequency Electromagnetic Waves on Unshielded Twisted Copper Pairs"

Ergin Dinc[1,*], Syed Sheheryar Bukhari[1], Anas Al Rawi[1,2], and Eloy de Lera Acedo[1]

[1]Dept. of Physics, Cavendish Laboratory, University of Cambridge, CB3 0HE, Cambridge, UK.
[2]BT labs, Adastral Park, Martlesham Heath, IP5 3RE, UK.
[*]ed502@cantab.ac.uk

ABSTRACT

This document includes the supplementary results for ‘High-Frequency Electromagnetic Waves on Unshielded Twisted Pairs: Upper Bound on Carrier Frequency". The list of the supplementary notes follows as
1- Numerical Simulation Environment
2- DM Launcher Design
3- Comparison of Simulation With and Without Dielectric
4- Measurements in anechoic laboratory environment
5- Radiation Measurements and Simulations
6- Bending TPs
7- Multiple TPs
8- Loss/m Results with Cut-back Simulations

Supplementary Note 1. Numerical Simulation Environment

Supplementary Figure 1 shows the numerical simulation environment. S-parameter results are generated with the time-domain solver of CST. The time-domain solver uses finite-difference time-domain techniques and especially efficient in investigating broadband response. TP are built by defining helical structures. The connection between the launcher and TP has a linear tapering and the length of this section is 10mm as seen in Supplementary Figure 1. This tapering is added not to cause any significant reflection. The distance between pairs at the cross-section of the DM launcher (g) is 2.18mm. There is a 2.5mm bonding cable between the pairs and traces on the DM launcher. This bonding wire is added to model the solder that is used in the experiments. The boundaries of the simulation are selected as “Open", which is equivalent to perfectly matched layer in other numerical tools. The minimum distance between the structure and the bounding box is selected as 300mm, which is around 5 wavelengths at 5GHz (the lowest radiation frequency observed in the simulations). The background material is assumed as vacuum. All of the simulations are stopped when the remaining field energy in the system is lower than -50dB.

In the simulations, copper is utilised for all conductors (5.8×10^7S/m). An initial narrow range is determined for the properties of dielectrics based on the available literature. Then, we selected the parameters giving the best match with our measurement results. We compare the results for the back-to-back launchers, 0.5m TP between launchers and 1m TP between launchers. Therefore, the selected values are validated with various measurement. The high consistency between the simulation results and measurements validates our methodology. According to the references[1,2], the following initial range is determined for FR-4: $\varepsilon_r = 4.3$-4.4, $\tan\delta = 0.021$-0.025. The final FR-4 parameters for the simulations are determined based on the back-to-back launcher measurement presented in Figure 5 in the main document and the following parameters are selected for FR-4: $\varepsilon_r = 4.3$, $\tan\delta = 0.021$. For PVC, the initial range for the dielectric properties is selected as $\varepsilon_r = 2.6$-2.9, $\tan\delta = 0.08$-0.01 based on[3,4]. The final values for PVC are determined by using measurements for 0.5m and 1m TPs and the following parameters are selected for the simulations: $\varepsilon_r = 2.7$, $\tan\delta = 0.01$.

DM launcher is excited with discrete face port in the simulations. Discrete ports in CST is used as lumped source element and often used while investigating radiation pattern of antennas. Discrete port is specifically selected for our simulations. In the case of waveguide ports, since micro-strip line is an inhomogeneous structure, the defined port needs to be at least 5-7 times larger than the thickness of the substrate. This is required for accurate port mode calculations in the waveguide ports; however, this waveguide port becomes ineffective if there is a radiating structure nearby because the port tries to include this radiation in the S-parameter calculations. We observe this effect when we compared our simulation and experiment results. This problem can be easily alleviated by using discrete face ports with 50Ω impedance. In this way, we observe highly consistent results

between the numerical simulations and measurements as shown in Figure 7 of the main document.

Supplementary Note 2. DM Launcher Design

The dimensions of the DM launcher can be seen in Supplementary Figure 2. Both double wire and narrow-end of the launcher support quasi-TEM modes as seen in Supplementary Figure 2(c,d). Hence, the supported mode at the end of the DM launcher and double wire are very similar. That's why, it is possible to achieve high launching efficiencies with the proposed microwave balun. We have used 50Ω SMA connector to excite the launcher. Therefore, the top trace of the microstrip line is selected as 3mm. The bottom layer of the microstrip line is selected as 28.8mm, which is 9-10 times of the top layer. Both top and bottom traces have a raised-cosine taper in order to reduce reflections. The length of the taper and β of the raised-cosine function is critical for the reflections from the launcher. We have simulated wide range of values for these parameters and chose the length of 100mm and $\beta = 0.08$. The length is especially important for the low-frequency response of the launcher because for lower wavelengths the taper is not as smooth as for the high frequencies due to the lower wavelengths. However, we need to choose a practical size and 100mm is selected for our measurements. The design of the taper can be optimised depending on the application and intended carrier frequency range. In this paper, the launcher is designed just to observe the radiation effect; hence, the launcher is designed to perform well around 5-10GHz. The linearity of the transmission response of the launcher in this frequency range, on the log-scale, makes it easier to detect the radiation. The width of the launcher is selected as 50mm, which is an arbitrary choice as any dimension higher than the metal trace at the bottom is acceptable. The dimension of the narrow-end of the launcher is already explained in the main document - "Design Guidelines for Differential Mode Launcher and Simulation Environment" section.

Note that, the DM launcher is designed by assuming that it only supports the dominant quasi-TEM mode. This is a valid assumption for the frequency range of interest in our work (<12GHz). If this design is utilised for higher frequency ranges without any modification, the DM launcher is likely to support higher order modes and the launching efficiency will be significantly lower due to mode conversion within the launcher. The cut-off frequency of the first higher order mode in microstrip lines are given in[5]. According to this reference, as a rule of thumb, the operation frequency of microstrip line should be much lower than a frequency whose wavelength is ten times of the thickness of the substrate. For our design, the first higher order mode is expected to appear at around 31GHz which is much higher than our frequency range of interest. Hence, a designer should be careful about avoiding higher order modes while designing a DM launcher for different frequency ranges. The operating frequency of DM launcher can be increased by simply using a substrate with lower thickness.

Supplementary Note 3. Comparison of Simulation With and Without Dielectric

Supplementary Figure 3 shows the comparison of the numerical simulations with and without dielectric coating around TP. S_{21} results with dielectric has higher losses as the dielectric introduces additional losses. In addition, the radiation frequency shifts to lower frequencies when there is a dielectric around TP. This is also an expected result because the dielectric coating causes higher propagation constant for the supported modes and as a result the radiation frequency decreases. If higher dielectric constants are used for the coating, the radiation frequency would move to even lower frequencies. Therefore, the designers should be careful about the dielectric properties of the wires that are used in the copper network while choosing the frequency spectrum because different wires may have different radiation frequency even though twist pitch length is unchanged.

Supplementary Note 4. Measurements in Chamber

To prove that there is no radiated energy between transmitter and receiver, we built an anechoic laboratory environment around one of the DM launcher as presented in Supplementary Figure 4(b). Carbon-loaded polyethylene foam tiles are used for absorbing any radiation from DM launcher, such that we can make sure that all of the received power is coming from the guided mode on TP. The custom build anechoic chamber includes a mix of tiles with thickness of 120mm and 150mm[6]. The used tiles have a 20dB-50dB absorption in the frequency range of 1-30GHz range. Supplementary Figure 4 includes the S_{11} and S_{21} measurement and simulation results for a 1m twisted pair (Wire 1 - $p = 25$-28mm). The simulations are performed for $p = 25$mm. There is only a slight decrease in the S_{21} curves when the tiles are present. This is an expected result as the wire is passing through the tiles and the wire is in contact with a very high loss material. Hence, we can conclude that there is no transmission between the transmitter and receiver in the form of radiation and all of the received power is coming from the guided wave on TP.

We also performed measurements for DM launchers with no wire between them. Supplementary Figure 5 includes the measurement results for this case. As expected, this case is associated with extremely low S_{21} values. This is another proof of the lack of radiative power transfer between the transmitter and receiver.

Supplementary Note 5. Radiation Measurements and Simulations

The radiation measurements were performed in order to practically demonstrate that the main source of power loss is radiation. In addition, we showed that the direction of the radiation is towards the backfire as predicted with the leaky-wave antenna theory in Figure 3 of the main paper. The sketch of the measurement can be seen in Supplementary Figure 6(a). The directional antenna is placed between the DM launchers in forward or backward direction. Only one of the launchers is excited, while the other one is terminated with a 50Ω load. An active log-periodic antenna (HyperLOG® 60100X) is used for measurements purchased from Aaronia AG (Germany). This antenna provides 45dBi gain in 680MHz to 10GHz spectrum. S_{22} of the antenna with and without preamplifier can be seen in Supplementary Figure 6(b). All the measurements are taken when the preamplifier is turned on. As noticed, the reflections from the antenna is very low at sub-5GHz spectrum. Although the reflections are a little higher after 5GHz, the values are still in an acceptable range for our measurements up to around 8GHz.

Supplementary Figure 6(c) includes the measurements for the forward and backward radiation when port 1 is connected to one of the launchers. As noticed, there is an increased signal levels in the backward direction radiation measurements starting from 5GHz, which is the radiation frequency estimated in the Figure 7(a) of the main document. This result proves that the direction of the radiation is consistent with our theory. In addition, the starting frequency of this radiation shifts to higher frequencies as the twist pitch lengths are lower as presented in Supplementary Figure 6(d-f). Since the far-field radiation signal that we tried to measure is very weak, the antenna needs to be placed in the close proximity of the TP. However, this causes the wire to be placed in the near-field of the antenna for lower frequencies (below 2GHz) thereby the result in this frequency range cannot be interpreted as far-field results. As a result, we also observe some radiation lower than 2GHz. The measured radiation starts to decline after 5GHz. There are two main reasons for this: (1) increased reflections of the directional antenna (see Supplementary Figure 6(b)) and (2) increasing path-loss due to higher carrier frequencies. However, this experiment well-justifies the direction of propagation and shift of the radiation frequency with the twist length. We can conclude that the measured radiation from TP is consistent with the theory developed in the main document.

After proving the presence of the radiation, it is also critical to demonstrate that the radiation is the dominant source of loss for the observed reduction in the S_{21} levels. Other possible explanations for this reduction in the received signal levels might be resonating modes and conversion of the propagating power to these modes. In order to justify our claims, we designed a simulation setup with the designed DM launcher and a TP wire with 15mm twist pitch length and 0.5m length. All material parameters are utilised as in the previous simulations. Supplementary Figure 7(a) shows the S-parameters for this system and there is a significant reduction in the S_{21} levels after 7.8GHz (This simulation result was also presented in Figure 7 of the main document and this result is included in the supplementary information for comparison with the following results). To calculate losses due to radiation, metals and dielectrics, we placed several monitors to the simulation domain. Radiation monitors are placed from 5GHz to 10GHz with 0.5GHz intervals. Three different values for the distance between the simulation bounding box and the closest simulated structure (L_{Box}) are utilised. Different values are simulated to show that there is no impact from the interaction between the propagating fields in the wire and the open boundary conditions. Supplementary Figure 7(b) shows the amount of power absorbed by the open boundary conditions (equivalent of perfectly-matched-layer in other simulation softwares). As noticed, there is a more than 4-fold increase in the radiated power at 8GHz, which is consistent with the S-parameters. Furthermore, all three simulated bounding box lengths are converged to almost identical values and this shows the accuracy of the results. Supplementary Figure 7(c) shows the distribution of the simulated power. Let's assume that the port 1 is excited in the simulation with an input power of 0.5W. Power outgoing from ports includes both transmitted power to port 2 and reflected power to port 1 during the simulation time. As seen in the figure, the power outgoing from the ports is decreasing with the frequency. This is an expected result as dielectric/metal losses are increasing with frequency and there is radiating power in the system as proved by previously discussed experiments and simulations. Note that FR-4 is used for the launchers thanks to its ease of fabrication, cost and availability, but it is a very lossy dielectric material[2]. The rapid fluctuations in the power is caused by the high S_{11} values of the DM launcher, which is also visible in the presented S-parameters in Supplementary Figure 7(a). As noticed in Supplementary Figure 7(c), the dielectric losses are increasing until 7.5GHz and remain constant after this frequency. Metal losses slightly decrease at 8GHz and remain nearly constant after that. Both of these trends are caused by the radiation as after 7.8GHz there is less amount of power on the wire interacting with metal and dielectric materials. These losses stay constant after the radiation frequency as the signal needs to pass through one of the DM launchers and excite the radiating mode on the TP. If there is an important amount of loss due to mode conversion and resonating modes, we need to observe an increase in dielectric and metal losses as well. In the end, the simulation results prove that the dominant source for the observed effect is the radiation due to the periodicity of the wire as predicted with our theory.

Supplementary Note 6. Bending TPs

In this supplementary note, the presence of radiation loss in TPs are investigated in more practical scenarios. It is important to show that this effect exists when the wire is bended as this is a highly likely situation in the real-world deployments.

Supplementary Figure 8 shows the S_{11} and S_{21} results for a 1m straight and 90° bended wire. As can be seen in Supplementary Figure 8(b), the bend has no effect on the radiation effect.

Supplementary Note 7. Multiple TPs

Supplementary Figure 9 presents the simulation and measurement results for multiple TPs. 0.5m TPs are utilised with the following characteristics: Wire 1 - p = 25-28mm (25mm is used in the simulation) and Wire 3 - p = 15-17mm (15mm is used in the simulation). As noticed in Supplementary Figure 9(d,e), both wires show the radiation effect at the frequency, which can be calculated with our proposed technique. This is a very important result because the modern ethernet cables CAT 5-8 consist of TPs having slightly different twist pitch lengths in order to lower cross-talk between wires. Therefore, the upper-bound in the carrier frequency needs to be calculated for each pair or the one with the highest twist pitch length in order to make sure that the system does not operate above the radiation frequency. The measurements are performed with a two-port VNA. All the possible two port combinations were measured one by one for the multiple pair measurements. Unmeasured ports are terminated with a 50Ω loads to avoid reflections

Supplementary Note 8. Loss/m Results with Cut-back Simulations

Supplementary Figure 10 includes the loss/m results for the differential mode on TP for various twist pitch lengths. To calculate loss/m results, we performed numerical simulations for 1m and 2m wire lengths in CST as can be seen in Supplementary Figure 10(a,b), respectively. Then, we subtracted these results from each other to find the loss/m results for the DM on TP. The loss/m results are shown in Supplementary Figure 10(c) and Supplementary Figure 10(d) is the zoomed version of Supplementary Figure 10(c). When the frequency is higher than the radiation frequency, the loss/m follow a random behaviour. This is expected as some of the power goes to the radiation and loss/m at these frequencies is not a meaningful metric. However, if we compare the results below 5GHz at which none of the wires are radiating, we can see that lower twist pitch lengths introduces higher losses. This is expected as for fixed length wires the one with lower twist pitch length has more copper and dielectric. Both of them are introducing losses and creates this effect. Straight double wire case is the upper-bound for loss/m for TP and included in the results as a comparison.

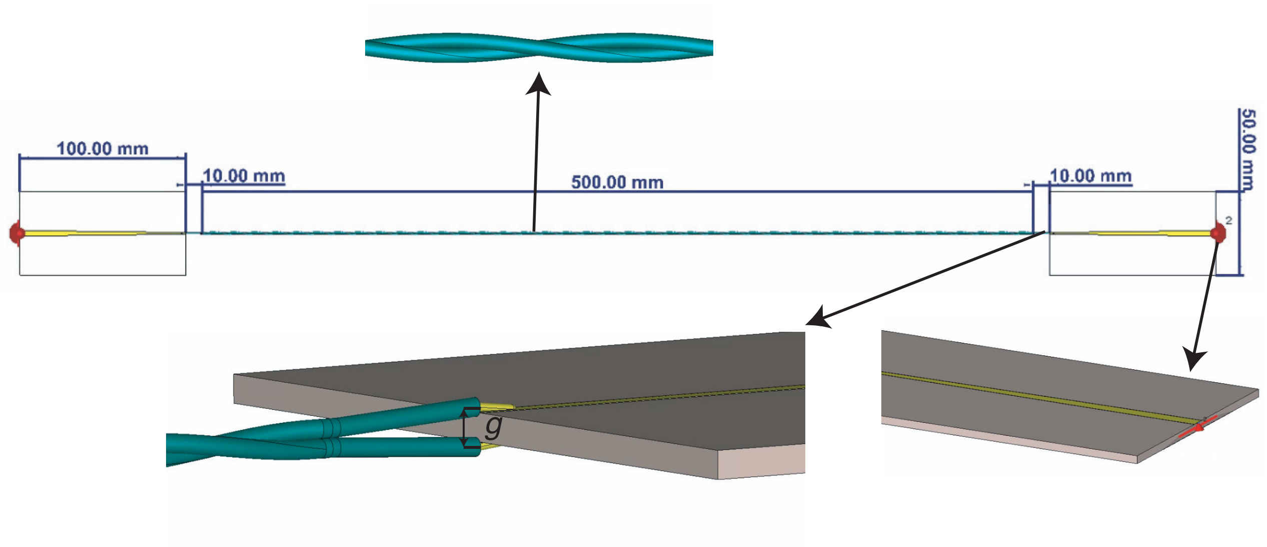

Supplementary Figure 1. Numerical Simulation Environment. Twisted pair is connected to the DM launcher with a 10mm untwisted section. Red arrows are the discrete port in CST. g is the separation between pairs at the DM launcher narrow-end.

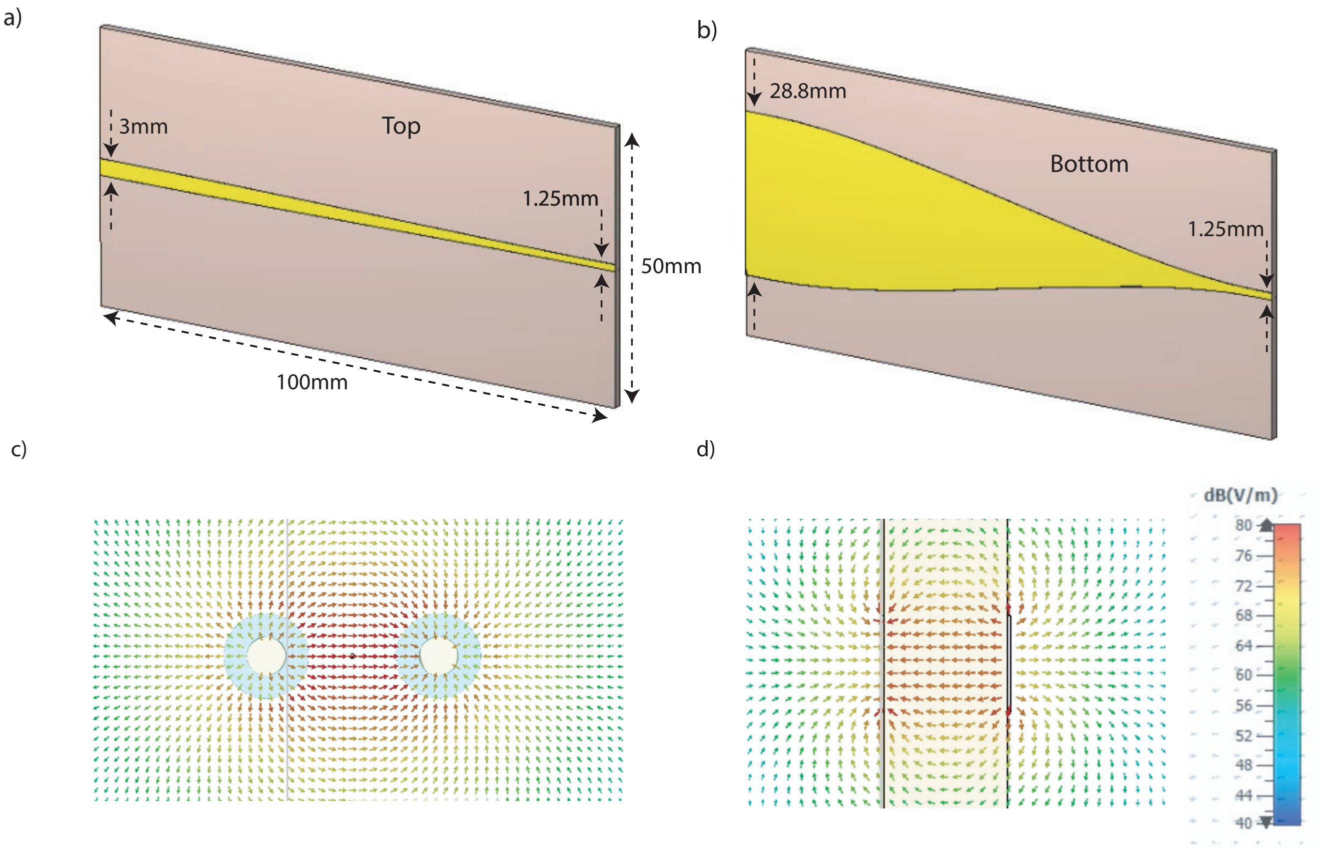

Supplementary Figure 2. Design of DM launcher (a) top and (b) bottom. (c) E-field of the quasi-TEM mode on double wire at 5GHz. (d) E-field of the quasi-TEM mode on the narrow-end of the launcher at 5GHz. The same colour scaling is used for (c) and (d).

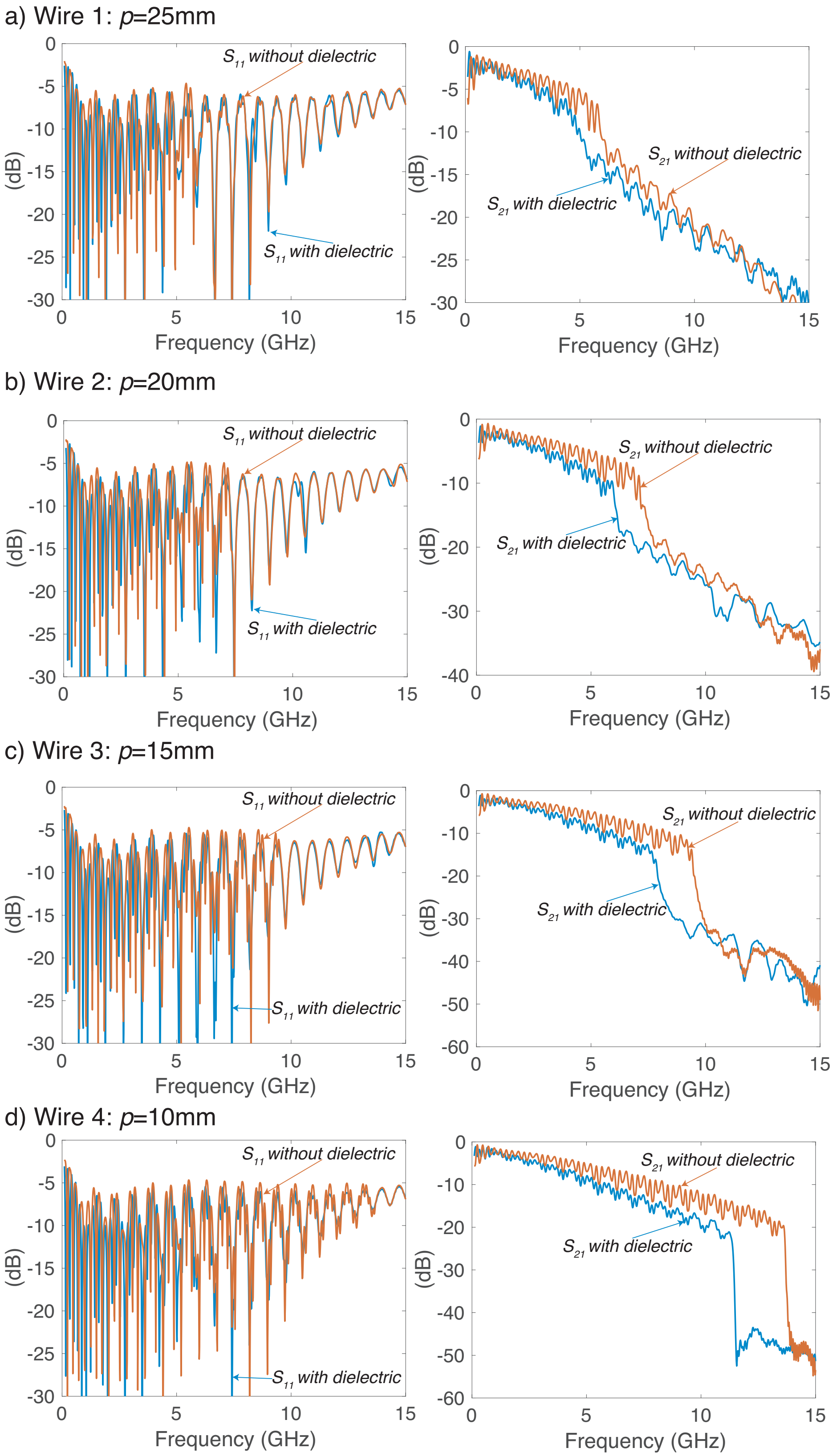

Supplementary Figure 3. Comparison of TPs with and without dielectric coating. Numerical S-parameters of the end-to-end system with and without dielectric coating around TP for different twist lengths (Wire length: 0.5m wires). p is the twist pitch length.

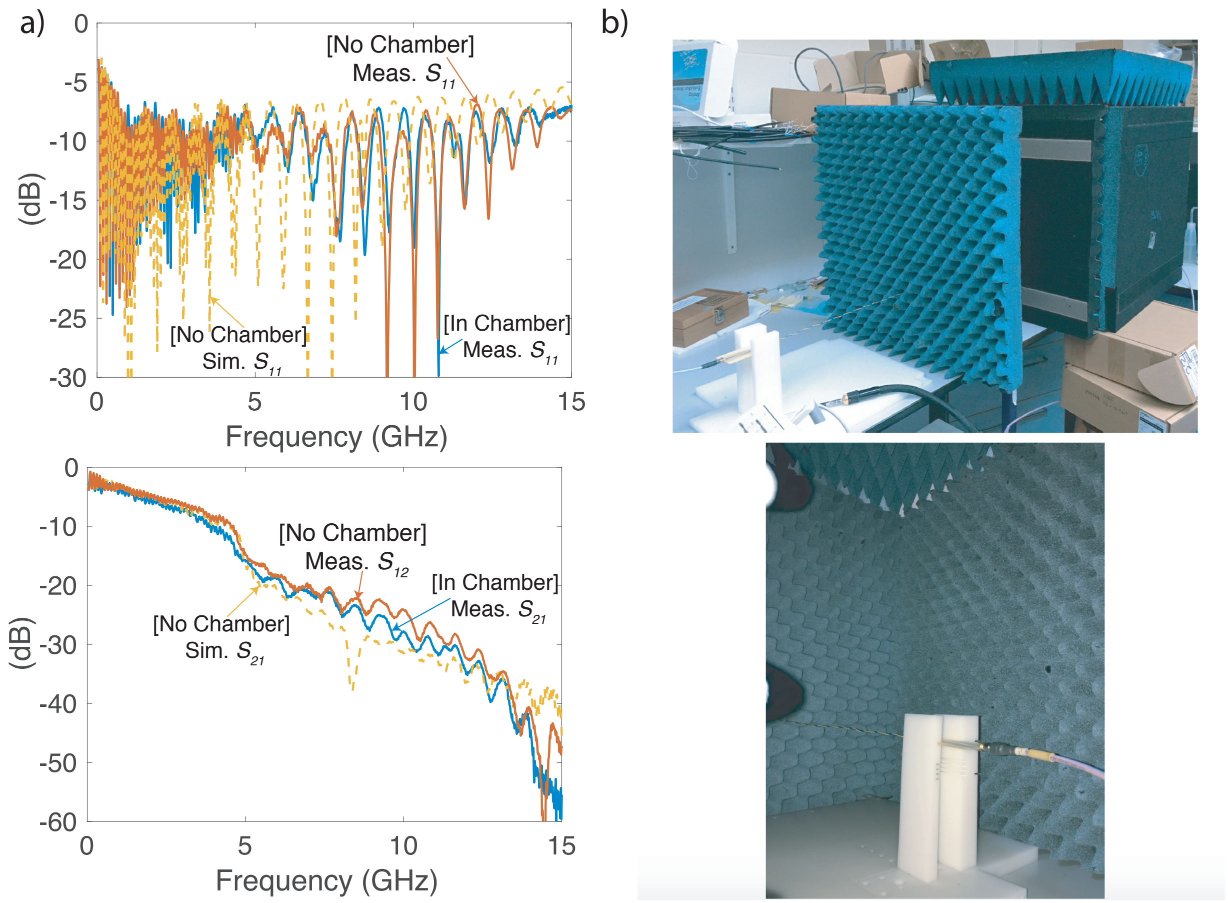

Supplementary Figure 4. Results in anechoic laboratory environment. Measurement and simulations results with and without the anechoic chamber tiles around the differential mode launcher for 1m Wire 1 - $p = 25$-28mm. a) S_{11} and S_{21} results. S_{21} results are affected by the presence of the chamber, but this change is due to the wire being in touch with the tiles. Therefore, we can conclude that the received signal is not due to the radiation. b) Photo of the measurement setup.

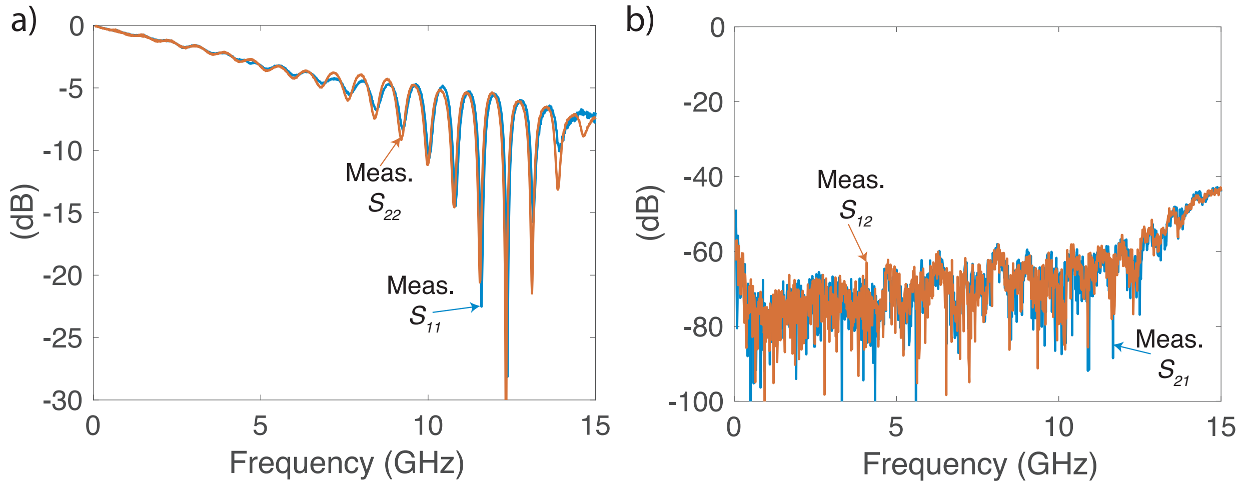

Supplementary Figure 5. Results for no wire between launchers. Measurement results with no transmission line between differential mode launchers. a) S_{11} and b) S_{21} results. This measurement results show that there is no power transfer between one differential mode launcher to the other in terms of radiation.

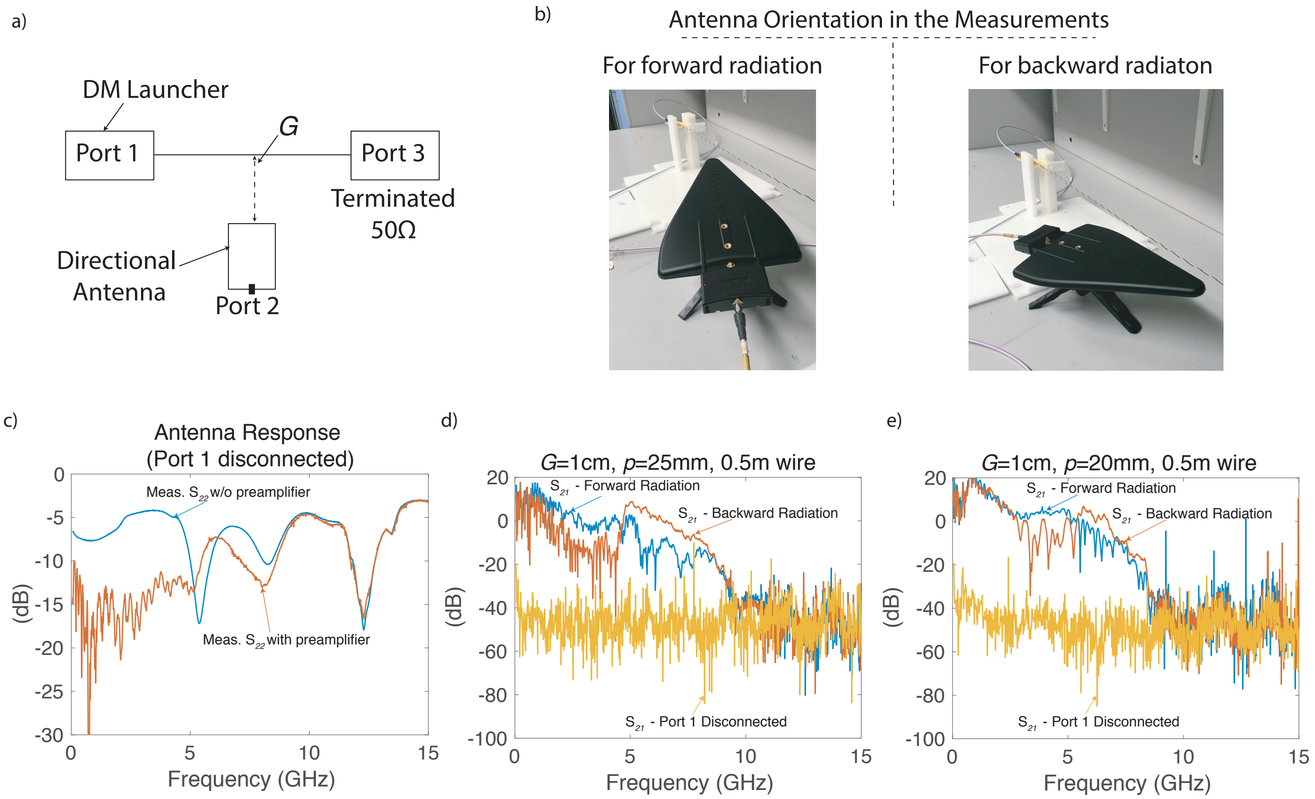

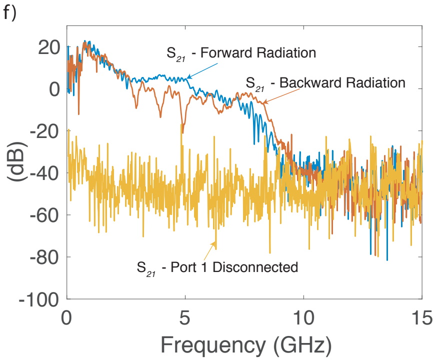

g)

Twist Length mm	*Radiation Frequency GHz (Based on Figure 7 of the main document)*	*Radiation Start Frequency GHz (From Antenna Measurements)*
25	*4.8*	4.78
20	*5.9*	5.7
15	*7.8*	7.4
10	*11.1*	-

Supplementary Figure 6. Radiation measurements. (a) A sketch of the measurement setup. DM launcher on the right is excited with Port 1 of the VNA and Port 2 of the VNA is connected to the directional antenna. The other DM launcher is terminated with a 50Ω load to avoid reflections. G is the distance of the antenna from TP. (b) antenna orientations for forward and backward radiation measurements. For forward radiation the antenna is pointed to the excited DM launcher and for backward measurement vice versa. (c) Reflection measurements of the antenna only. Port 1 is disconnected. (d) Radiation measurements for Wire 1 - $p = 25$-28mm, 0.5m wire, where p is the twist pitch length, (e) Radiation measurements for Wire 2 - $p = 20$-22mm, 0.5m wire, (f) Radiation measurements for Wire 3 - $p = 15$-17mm, 0.5m wire, and (g) radiation frequency results (simulations) determined from Figure 7 of the main document and radiation measured with the antenna. The difference between results is caused by the nonuniform twist of real world cables.

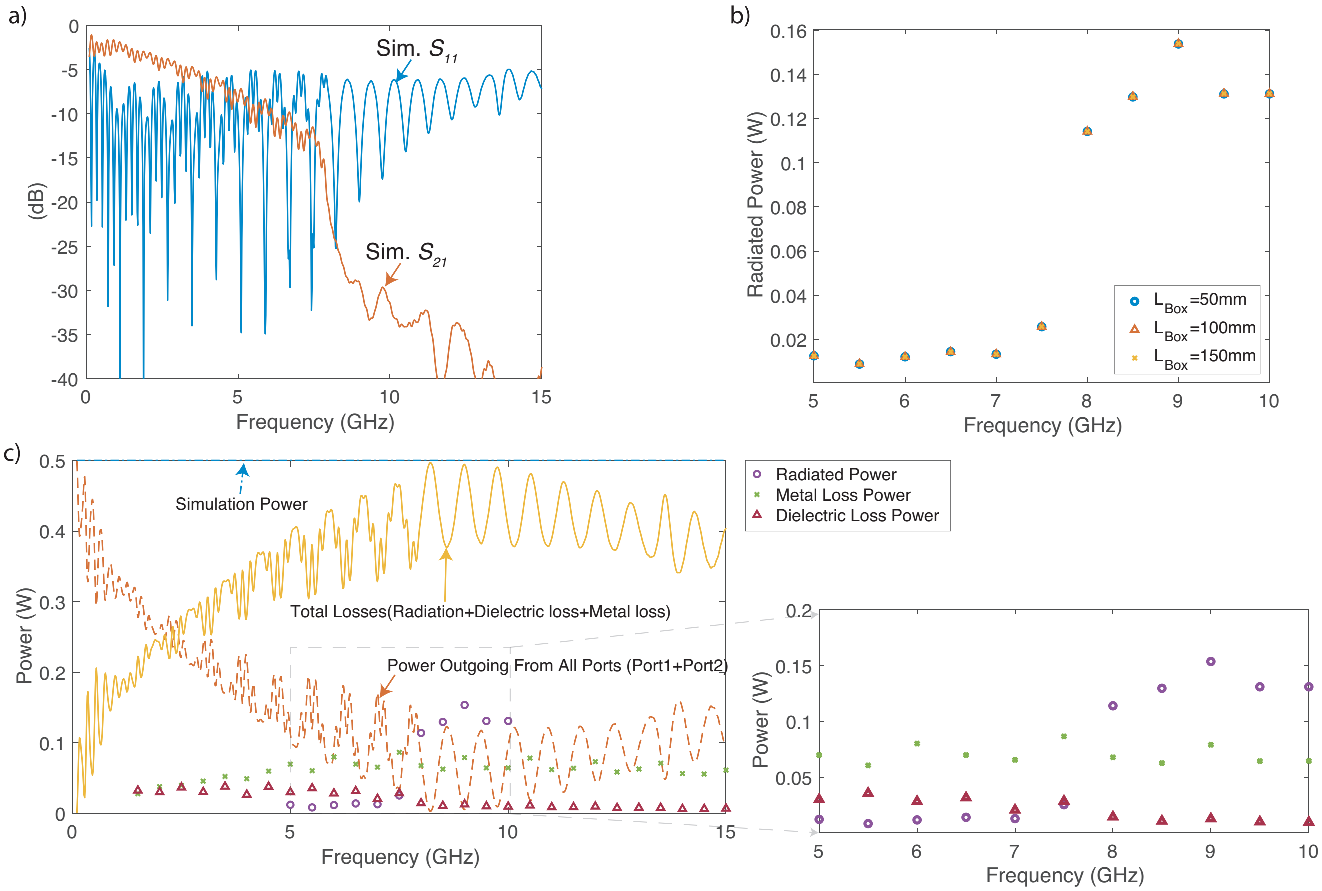

Supplementary Figure 7. Radiation simulations. (a) S-parameters for a TP with twist pitch length 15mm and 0.5m length (This is the same result presented in Fig 7(c) of the main document). b) Radiated power results from CST for three different bounding box values, where L_{Box} is the distance of the open boundary from the closest simulated structure in the simulation domain. All of the values resulted in almost the same radiated power levels and there is a significant increase in the radiation at 8GHz, which is consistent with the S-parameters. c) Simulation power results including the losses. Power outgoing from all ports include the power received by both Port 1 and Port 2 during the simulation time. Total losses include radiation as well as metal and dielectric losses. After 7.8GHz, dielectric losses mostly remain at a constant level, but metal losses first decrease and then stabilise at a lower level. This shows that there is no significant amount of resonating power in the structure; therefore, this result proves that the dominant source for the observed effect is the radiation due to the periodicity of the wire. The zoomed version of the losses is included for showing the sudden increase in the radiated power after the radiation frequency.

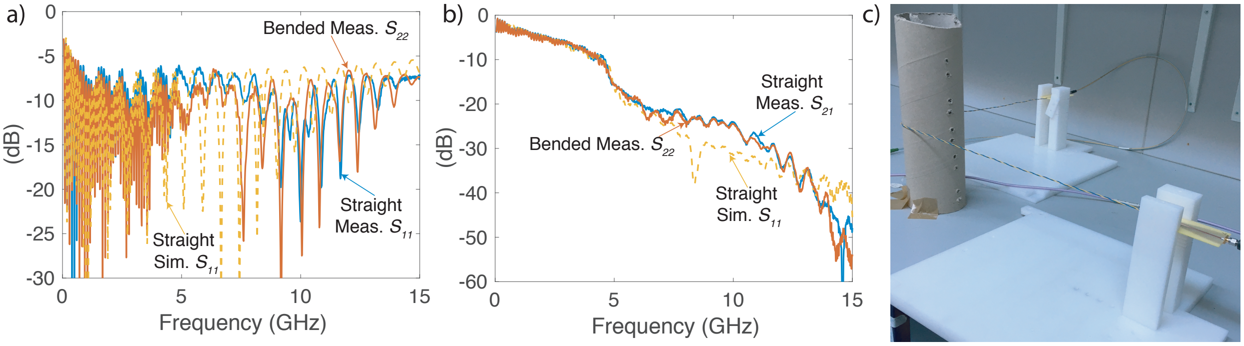

Supplementary Figure 8. Bended wire measurements. Measurement and simulations results for demonstrating the existence of the radiation effect when the cable is bended. Wire length 1m in all cases. a) S_{11}. b) S_{21}: Results clearly suggest that the radiation effect exist when the cable is bended, and c) Photo of the measurement setup. The bending radius is 5.5cm.

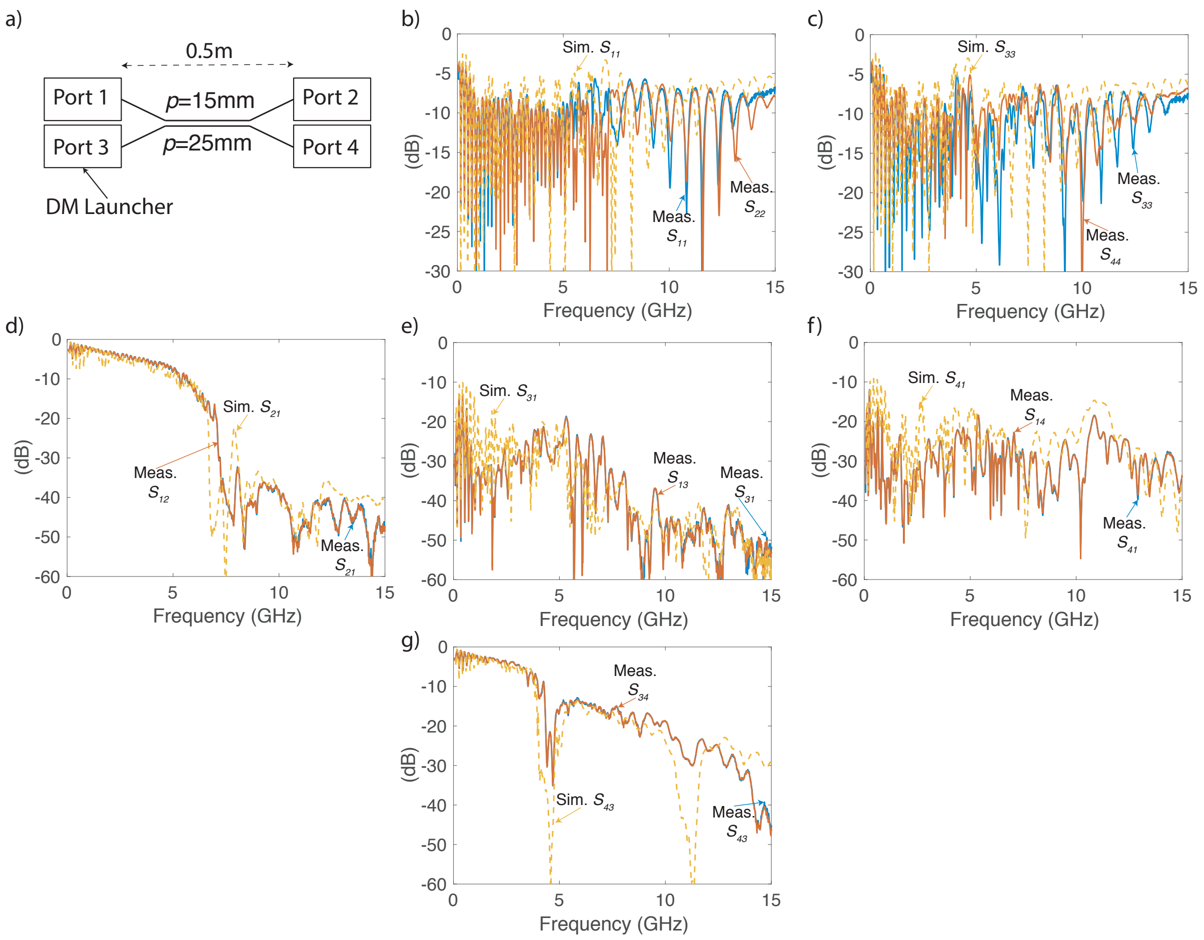

Supplementary Figure 9. Multiple twisted pairs Measurement and simulations results for multiple twisted pairs, where p is the twist pitch length. a) Block diagram of the simulated and measured setup, b) S_{11} and S_{22} results, c) S_{33} and S_{44} results, d) S_{12} and S_{12} results, e) S_{13} and S_{31} results, f) S_{41} and S_{14} results, g) S_{43} and S_{34} results.

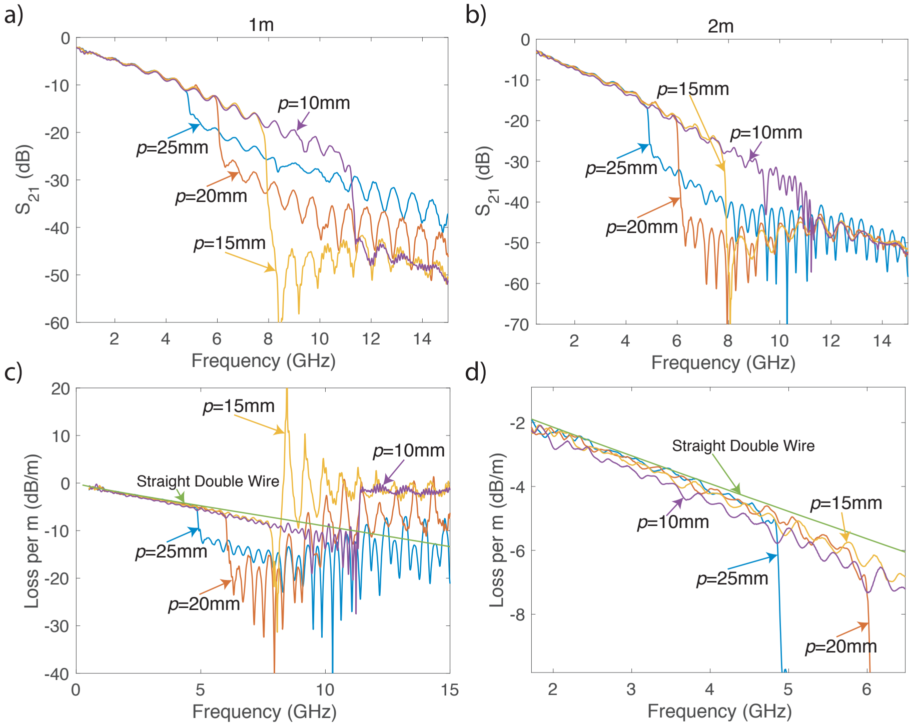

Supplementary Figure 10. **Loss/m results derived by cut-back simulations.** a) S_{21} results for 1m cable, where *p* is the twist pitch length, b) S_{21} results for 2m, c) Loss/m results derived by subtracting 1m and 2m results, and d) zoomed version of the plot in (c).

Supplementary References

1. Aguilar, J. R., Beadle, M., Thompson, P. T. & Shelley, M. W. The microwave and rf characteristics of fr4 substrates. In *IEE Colloquium on Low Cost Antenna Technology (Ref. No. 1998/206)*, 2/1–2/6 (1998).

2. Cai, L. & Zeng, Y. Extraction of FR4 dielectric properties in microwave bands using ABCD matrix. In *2020 IEEE MTT-S International Wireless Symposium (IWS)*, 1–3, DOI: 10.1109/IWS49314.2020.9360178 (2020).

3. Ghodgaonkar, D., Varadan, V. & Varadan, V. A free-space method for measurement of dielectric constants and loss tangents at microwave frequencies. *IEEE Transactions on Instrumentation Meas.* **38**, 789–793, DOI: 10.1109/19.32194 (1989).

4. Bur, A. J. Dielectric properties of polymers at microwave frequencies: a review. *Polymer* **26**, 963–977, DOI: https://doi.org/10.1016/0032-3861(85)90216-2 (1985).

5. Bahl, I. J., Trivedi, D. K. *A Designer's Guide to Microstrip Line* (Microwaves, 1977), 174–182.

6. TDK. Microwave anechoic chambers. [Online]. Available at:https://www.tdkrfsolutions.tdk.com/images/uploads/data-sheets/TDK-IS-Absorber-Series_032021.pdf [Accessed on 12/01/2022].
